# Regulation of ErbB2 Receptor Status by the Proteasomal DUB POH1

**DOI:** 10.1371/journal.pone.0005544

**Published:** 2009-05-14

**Authors:** Han Liu, Richard Buus, Michael J. Clague, Sylvie Urbé

**Affiliations:** Physiological Laboratory, School of Biomedical Sciences, Liverpool, United Kingdom; University of Birmingham, United Kingdom

## Abstract

Understanding the factors, which control ErbB2 and EGF receptor (EGFR) status in cells is likely to inform future therapeutic approaches directed at these potent oncogenes. ErbB2 is resistant to stimulus-induced degradation and high levels of over-expression can inhibit EGF receptor down-regulation. We now show that for HeLa cells expressing similar numbers of EGFR and ErbB2, EGFR down-regulation is efficient and insensitive to reduction of ErbB2 levels. Deubiquitinating enzymes (DUBs) may extend protein half-lives by rescuing ubiquitinated substrates from proteasomal degradation or from ubiquitin-dependent lysosomal sorting. Using a siRNA library directed at the full complement of human DUBs, we identified POH1 (also known as Rpn11 or PSMD14), a component of the proteasome lid, as a critical DUB controlling the apparent ErbB2 levels. Moreover, the effects on ErbB2 levels can be reproduced by administration of proteasomal inhibitors such as epoxomicin used at maximally tolerated doses. However, the extent of this apparent loss and specificity for ErbB2 versus EGFR could not be accounted for by changes in transcription or degradation rate. Further investigation revealed that cell surface ErbB2 levels are only mildly affected by POH1 knock-down and that the apparent loss can at least partially be explained by the accumulation of higher molecular weight ubiquitinated forms of ErbB2 that are detectable with an extracellular but not intracellular domain directed antibody. We propose that POH1 may deubiquitinate ErbB2 and that this activity is not necessarily coupled to proteasomal degradation.

## Introduction

The ErbB2/Her2 receptor is one of four members of the ErbB family of receptor tyrosine kinases (RTKs) [1], [2]. Its over-expression in breast cancers is associated with poor prognosis and malignancy. It is a high priority drug target, against which monoclonal antibodies (e.g. Herceptin) are used as a frontline therapy. The receptor possesses no ligand binding affinity and is only activated upon ligand-induced hetero-dimerisation with another family member, for example EGF Receptor (EGFR). Upon activation, most RTKs are down-regulated through Cbl-dependent ubiquitination and ubiquitin-dependent sorting to the lysosome [3]. Uniquely amongst the ErbB family, ErbB2 is endocytosis defective, with the consequence that its over-expression may also interfere with the down-regulation of ErbB family binding partners [4], [5], [6], [7]. To date the influence of ErbB2 on EGFR down-regulation has been studied by over-expression, but the inverse approach of ErbB2 knock-down has not been explored.

The ubiquitin system influences nearly all aspects of cell physiology [8]. It can determine protein stability, by promoting both proteasomal and lysosomal degradation, but also regulates transcription and translation. The hsp90 inhibitor Geldanamycin induces the down-regulation of ErbB2 [9]. Ubiquitination of the receptor becomes evident and proteasome inhibitors reverse Geldanamycin-induced degradation [10], [11], most likely indirectly by interfering with lysosomal trafficking of the receptor [12], [13], [14]. Ubiquitination can be reversed by the action of deubiquitinating enzymes (DUBs), of which there are around 85 active members falling into 5 major families [15]. These enzymes are emerging as attractive drug targets [16].

In this study we have identified a requirement for a DUB associated with the proteasomal 19S complex, POH1 (also known as Rpn11 or PSMD14), in the regulation of ErbB2 ubiquitination.

## Results

### Role of ErbB2 in EGF receptor down-regulation and signalling

**Figure 1:**
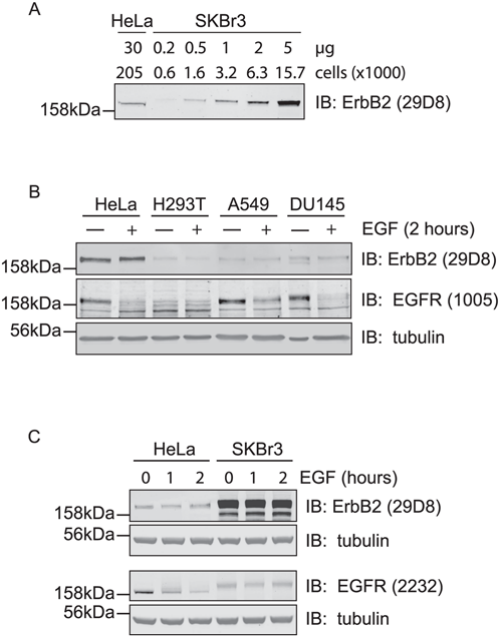
ErbB2 escapes EGF induced down-regulation. A, Comparison of ErbB2 receptor levels in HeLa and SKBr3 cells. Cell lysate samples corresponding to the indicated number of cells were separated by SDS-PAGE and immunoblotted with ErbB2 antibodies and IR800-coupled secondary antibodies. The relative amount of ErbB2 per cell was calculated based on Odyssey scans as discussed in Materials and Methods. B, HeLa, HEK293T (H293T), A549, and DU145 cells were stimulated with 100 ng/ml EGF for 2 hours and lysed in parallel with unstimulated cells. The lysate was subjected to SDS-PAGE and immunoblotting with EGFR, ErbB2, and tubulin antibodies. EGFR is down-regulated after 2 hours stimulation, but ErbB2 remains stable. C, HeLa and SKBr3 cells were treated with 100 ng/ml EGF for 1 or 2 hours and analysed by immunoblotting with EGFR and ErbB2 antibodies. No EGFR was detected in SKBr3 cells. Note that EGFR antibody (2232) cross-reacts with high levels of ErbB2 in SKBr3 cells.

It has been established that SKBr3 cells highly over-express ErbB2 (2.7×10^6^) [17] and that HeLa cells possess around 50,000 EGF receptors [18]. Using these estimates as benchmarks, we have extrapolated relative levels of receptors to other cell lines by quantitative immuno-blotting using an Odyssey Imaging system. Thus we can estimate the number of ErbB2 receptors on our HeLa cells to be in the order of 54,000 and the number of EGFRs on A549 cells as around 67,000 (Figure 1A and B). Following EGF stimulation, ErbB2 levels remained constant whilst EGFR levels declined over a 2 hours time period in HeLa, A549 and DU145 cells (Figure 1B). The degradation rate of EGFR between various cell lines did not correlate with reduced ErbB2 levels. Degradation of EGFR in A549 cells is incomplete after 2 hours, yet complete in HeLa cells, which have a higher ErbB2 to EGFR ratio by an order of magnitude (Figure 1B). We could not unambiguously detect EGFR in SKBR3 cells; the band seen by Western blotting with anti-EGFR antibodies is most likely due to minor cross reactivity with ErbB2, based on expression levels and molecular weight considerations (Figure 1C).

**Figure 2:**
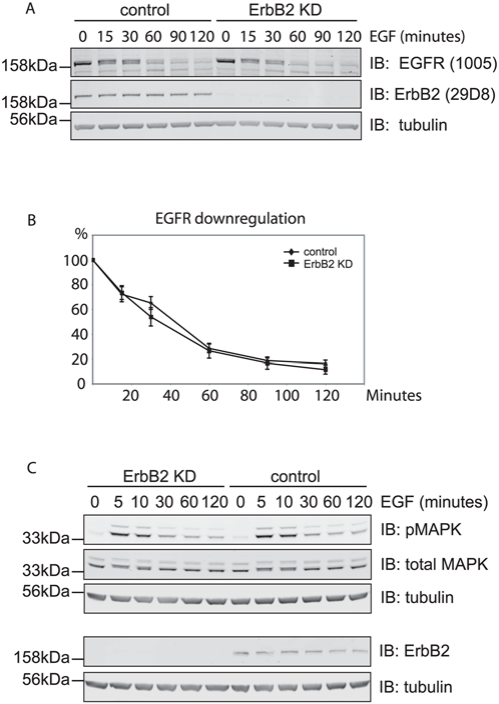
Effects of ErbB2 depletion on EGFR down-regulation and downstream signaling in HeLa cells. A. HeLa cells were treated with ErbB2 siRNA or oligofectamine transfection reagent (control) for 48 hours before stimulation with 100 ng/ml EGF for various time periods. Lysates were analysed by immunoblotting with EGFR and ErbB2 antibodies. B. quantitation of A showing EGFR down-regulation was not significantly affected (data averaged from 3 experiments). C. HeLa cells were treated as in A and incubated with 10 ng/ml EGF for different time periods and lysed. Lysate was analysed by immunoblotting with ErbB2, pMAPK, MAPK, and tubulin antibodies. Levels of total MAPK and pMAPK were not affected by ErbB2 knock-down.

Knock-down of ErbB2 had no effect on the EGFR degradation rate in HeLa cells (Figure 2A,B), nor did it influence MAP kinase (MAPK) signalling as evidenced by immunoblotting with anti-phospho-MAPK.

### Screening for DUBs controlling ErbB2 levels

**Figure 3:**
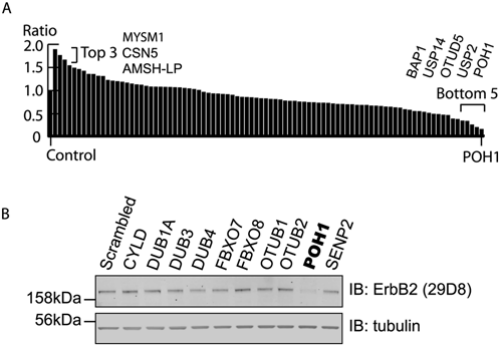
DUB screen for altered ErbB2 levels in HeLa cells. siRNA mediated knockdown of DUBs was carried out in HeLa cells (siRNA at 40 nM). Cells were lysed after 72 hours and analysed by immunoblotting with ErbB2 antibody. A. The amount of ErbB2 for each sample was normalized to tubulin and expressed relative to control (non-targeting oligo) for each DUB contained in the library. B. ErbB2 levels in cells treated with a sub-set of the siGenome DUB library.

**Figure 4:**
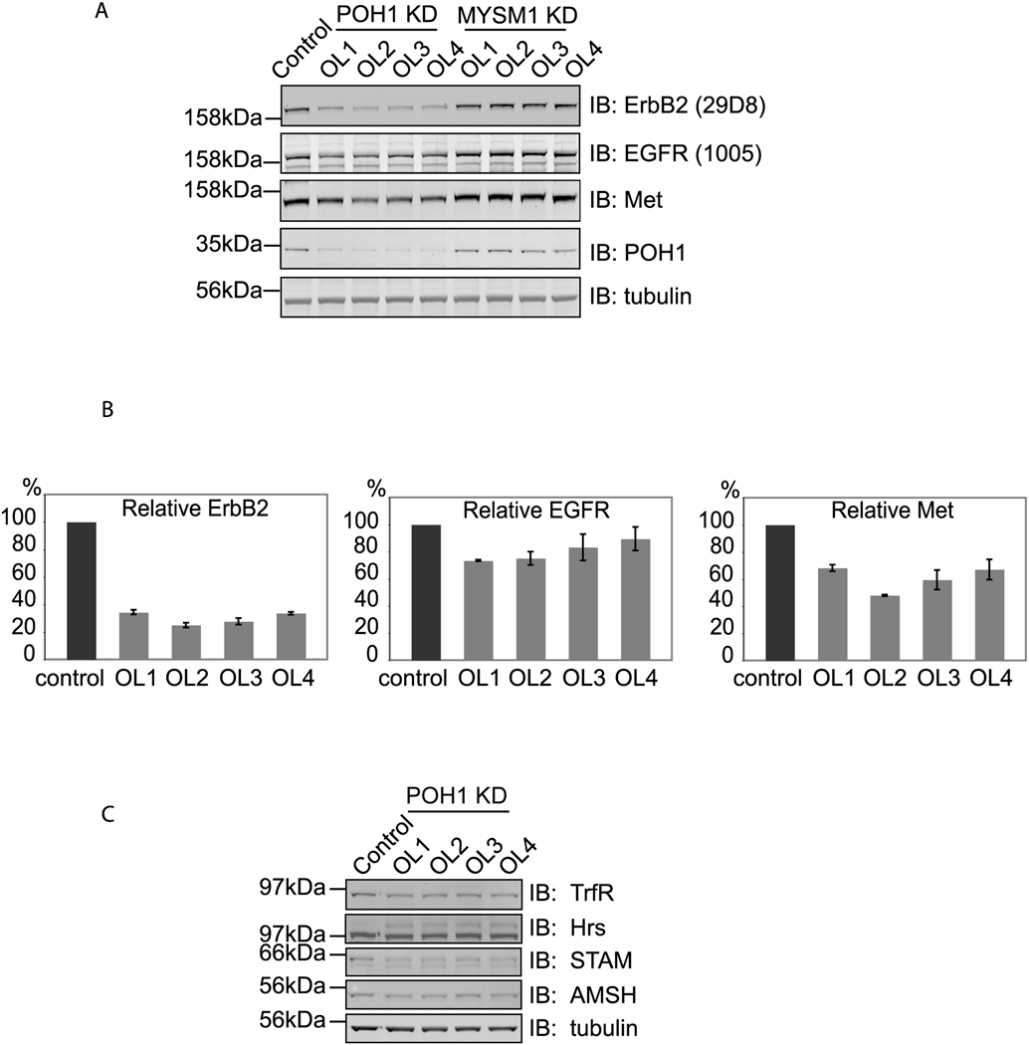
Multiple POH1 oligos down-regulate ErbB2. A. siRNA mediated knockdown (KD) of candidates was repeated with four individual On Target Plus oligos incubated with HeLa cells for 48 hours. The results for POH1 were consistent. The knockdown of other candidates (MYSM1, USP14, BAP1, etc) with four individual oligos failed to show significant effects on ErbB2 levels (results from MYSM1 shown as an example). Lysates were also probed with EGFR, Met, and tubulin antibodies. The effect of knock-down of POH1 on EGFR and Met levels was much less pronounced than for ErbB2. B. quantitation of ErbB2, EGFR, and Met receptors for POH1 knockdown cells (averaged from 3 experiments). C. HeLa cells were treated with POH1 siRNA (four individual oligos) or oligofectamine transfection reagent (control) for 48 hours before lysis and analysed by immunoblotting for Transferrin receptor (TfR), Hrs, STAM, AMSH, and tubulin. Knock-down of POH1 showed minor effects on the levels of these proteins.

We screened a siRNA (siGenome, Dharmacon) library for DUBs, which control the stability of ErbB2, using 29D8 monoclonal antibody, which recognises an epitope in the cytoplasmic domain. We then assembled our results into a rank order (Figure 3). The screen is conducted with a pool of 4 oligos for each DUB. We selected DUBs at extreme ends of the spectrum of ErbB2 levels for validation with individual On-Target Plus oligos. 3 DUBs MYSM1, CSN5 and AMSH-LP showed >1.5 fold increase in ErbB2 in our initial screen, but in no case could this be confirmed with On-Target Plus Oligos applied individually. BAP1, USP14, USP2, and POH1 all indicated substantial loss of ErbB2 in the initial screen. Only POH1 could be convincingly validated, showing significant decrease in ErbB2 with all four On-Target Plus oligos (Figure 4A). Whilst small effects on the levels of other RTKs EGFR and Met were also evident, this was most striking for ErbB2, suggesting some degree of selectivity (Figure 4A,B). No noticeable change was observed in the Coomassie Blue staining pattern following POH1 knock-down (not shown), nor in the levels of Transferrin receptor or the endosome associated proteins STAM and AMSH (Figure 4C). However, a higher molecular weight form of the ESCRT-0 component Hrs, was evident, which would be consistent with its mono-ubiquitination (Figure 4C) [19].

### Determining the mechanism of ErbB2 loss following POH1 knock-down

**Figure 5:**
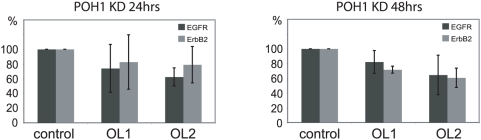
POH1 depletion does not differentially affect ErbB2 and EGFR transcription levels. HeLa cells were treated±POH1 siRNA (two individual oligos) for 24 or 48 hours (left and right panels respectively) before RNA was extracted. Levels of mRNA of EGFR and ErbB2 were calculated relative to actin mRNA. Graph shows qRT-PCR results averaged from 3 experiments. Error bars show standard deviation.

**Figure 6:**
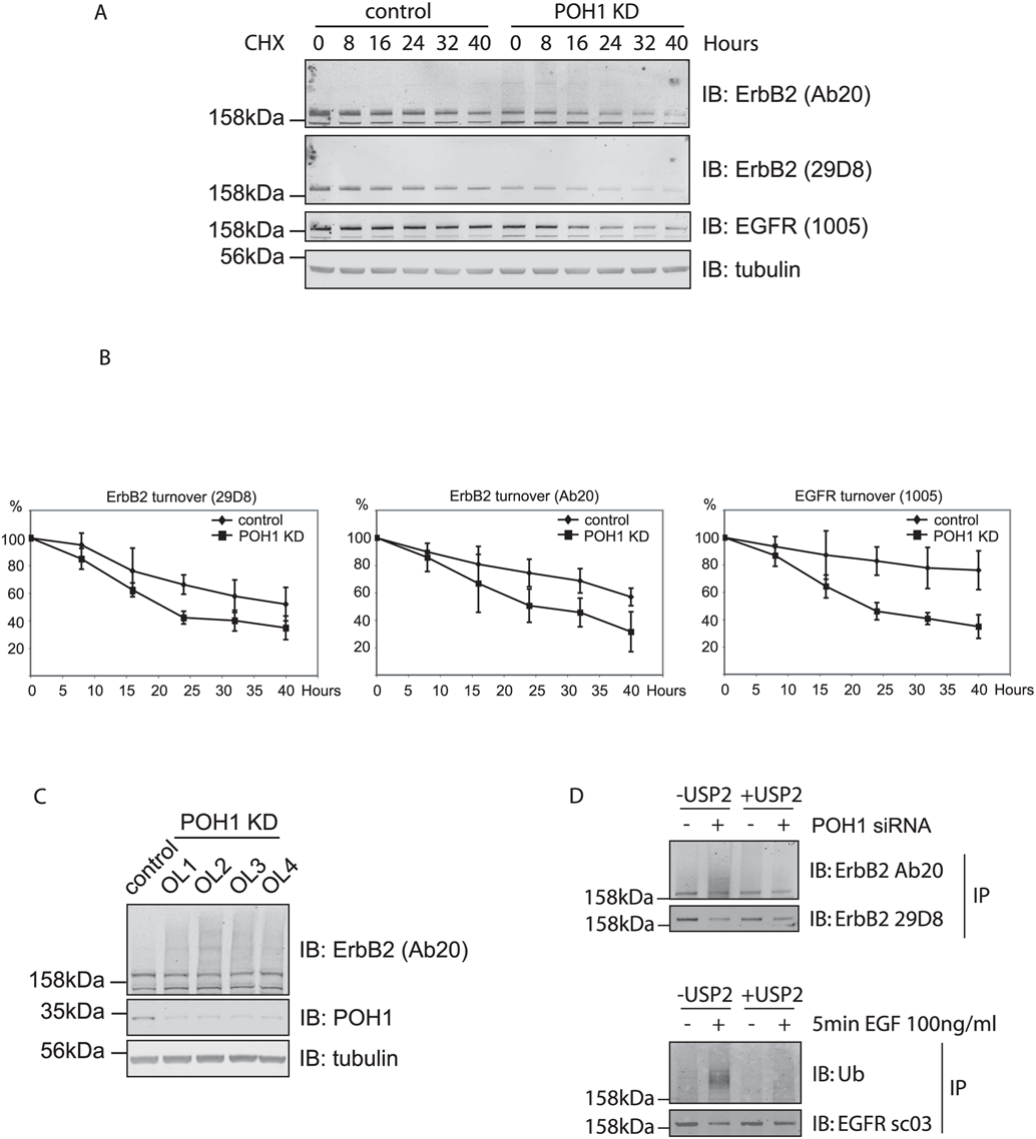
POH1 depletion and ErbB2 receptor turnover. A, HeLa cells were treated±POH1 siRNA for 48 hours before incubation with 10 µg/ml cycloheximide. Cells were lysed and analysed by immunoblotting with ErbB2 29D8 and Ab20 antibodies, which recognize intracellular and extracellular epitopes of ErbB2 respectively, EGFR, and tubulin antibodies. B, quantitation shows that both EGFR (by antibody 1005) and ErbB2 (by antibodies Ab20 and 29D8) are turned over more rapidly in POH1 knock-down cells (data averaged from 3 experiments). C. HeLa cells were treated with four On Target Plus oligos (POH1) or with oligofectamine alone for 72 hours before lysis with hot lysis buffer. A higher molecular weight ErbB2 “smear” was observed in all 4 knock-down samples. D The high molecular weight smear associated with ErbB2 immuno-reactivity is sensitive to treatment with a deubiquitinase (USP2). HeLa cells were treated with POH1 siRNA or oligofectamine for 48 hours before lysis in the presence of NEM. ErbB2 was immunoprecipitated and treated *in vitro* with USP2 catalytic domain (100 nM, 8 hours, 37°C). Samples were analyzed by immunoblotting with ErbB2 antibodies targeting extracellular (Ab20) and intracellular (29D8) domains. Note that the smear detected with Ab20 is lost upon USP2 treatment whilst detection with the intracellular domain antibody increases. As a control for USP2 DUB-activity, EGFR was immunoprecipitated from EGF-stimulated (5 min) HeLa cells and treated in vitro with USP2 catalytic domain before SDS-PAGE and western blotting with anti-Ubiquitin.

**Figure 7:**
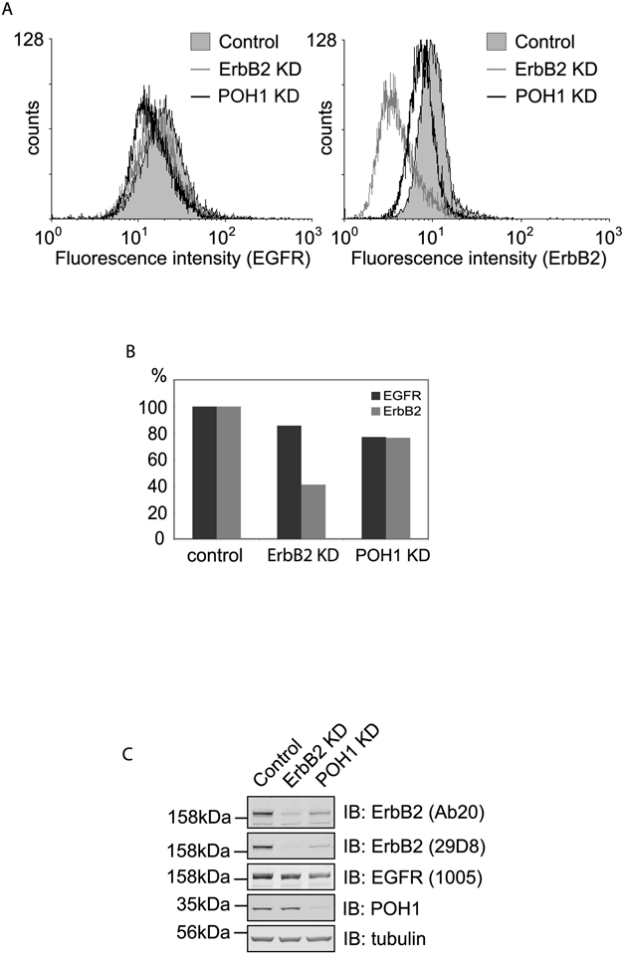
Cell surface levels of ErbB2 are relatively insensitive to POH1 knock-down. A, HeLa cells were treated with ErbB2, POH1 siRNA or oligofectamine alone for 48 hours before detachment with 2 mM EDTA. One million cells from each condition were labelled with FITC-conjugated ErbB2 and phycoerythrin (PE) conjugated EGFR antibodies and then analysed by flow cytometry. B, quantification of A shows relative amounts of fluorescence for each condition. C, samples from A were analysed by immunoblotting with ErbB2 (Ab20 and 29D8), EGFR, POH1, and tubulin antibodies.

What is the mechanism of this apparent ErbB2 down-regulation following POH1 knockdown? In principal this could reflect changes in transcription, translation or protein turnover. Determination of mRNA levels by RT-PCR revealed a slight decrease at the 48 hrs time-point, but this is not of the requisite magnitude (assuming linearity), nor does it exhibit selectivity for ErbB2 over EGFR (Figure 5). We next measured the rate of loss of ErbB2 and EGFR following a cycloheximide-induced block to translation. Knockdown of POH1 gave a modest increase in ErbB2 turnover, but significantly enhanced the down-regulation of EGFR (Figure 6A,B). Whilst interesting, these results cannot account for the preferential loss of ErbB2 at steady state. Levels of Hsp90, a factor known to control ErbB2 stability [11], were unchanged (not shown). Using an antibody against an extracellular epitope of ErbB2, Ab20, we obtained very similar results, however we noticed the presence of a faint, higher molecular weight smear specific to POH1 knock-down conditions (Figure 6A top panel) that is characteristic of ubiquitination. We verified that this phenomenon was reproduced by all four On-Target Plus oligos directed against ErbB2 (Figure 6C). Given our failure to find a substantial defect in ErbB2 transcription or protein degradation rates, we suspected this higher molecular weight form may represent ubiquitinated ErbB2 that accumulates upon POH1 knockdown and may be less easily detectable by western blotting due to its heterologous molecular weight. This effect may be further compounded by using an antibody directed against an intracellular epitope (29D8), which may conceivably be masked by this modification. Indeed, the high molecular weight smear detected with the extracellular antibody is susceptible to *in vitro* protease treatment with the catalytic domain of USP2, a non-specific DUB that can be used in a similar way to alkaline phosphatase treatment to query the phosphorylation status of a protein, and which is able to remove ubiqutin from activated EGFR, a well-established ubiqutinated protein (Figure 6D). Concomitantly, removal of ubiqutin by USP2 also leads to a partial recovery of the ErbB2 signal detected with the intracellular domain antibody, suggesting regeneration of an epitope that was previously masked by ubiquitin. A western blotting approach may thus lead us to misjudge the actual amount of ErbB2 expressed in these cells. Using fluorescently labelled anti-ErbB2 or anti-EGFR extracellular antibodies allowed us to measure cell surface ErbB2 and EGFR levels following POH1 knockdown by FACS analysis (Figure 7A,B). This approach showed only modest reductions in the levels of both receptors. When the same samples were probed by western blotting with anti-ErbB2 or anti-EGFR antibodies a highly significant and preferential loss of ErbB2 is observed, that is however more striking for the intracellular ErbB2 antibody (29D8) (Figure 7C).

### POH1 depletion and proteasome inhibition

**Figure 8:**
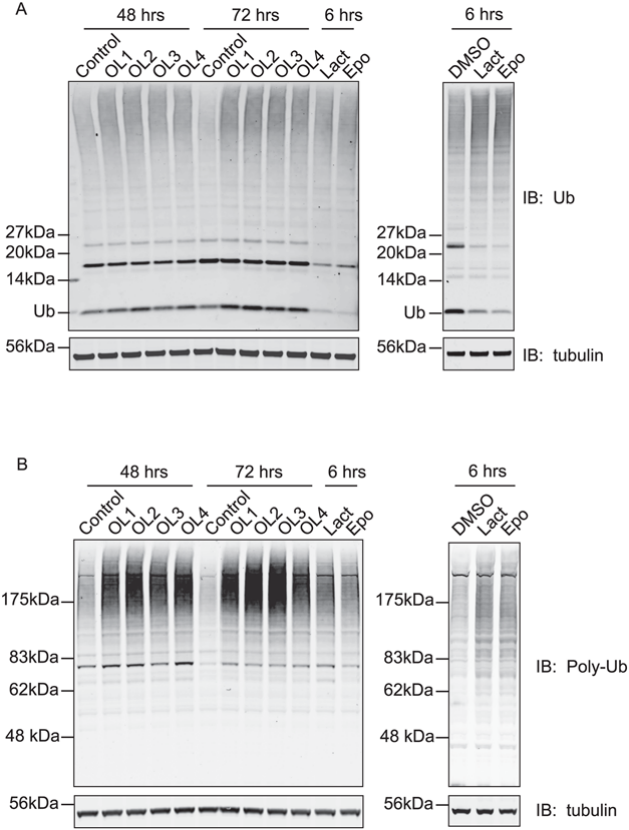
POH1 deletion increases levels of free ubiquitin and ubiquitinated proteins. HeLa cells were treated with POH1 siRNA (oligo 1–4) or control reagent for 48 or 72 hours before lysis with “hot lysis” SDS-buffer. Equal amounts of cell lysates were analysed by immunoblotting with A, polyclonal anti-ubiquitin (recognising ubiquitinated proteins and free ubiquitin) and B, FK1 monoclonal anti-ubiquitin (recognising only polyubiquitinated proteins). As with proteasome inhibitor treatment (Lact: lactacystin, 10 µM and Epo: epoxomicin, 10 µM or 1 µM left and right panels respectively), POH1 depletion caused an accumulation of ubiquitinated proteins in the cell, but in contrast to the inhibitors, which deplete free ubiquitin, levels of free ubiquitin were elevated.

**Figure 9:**
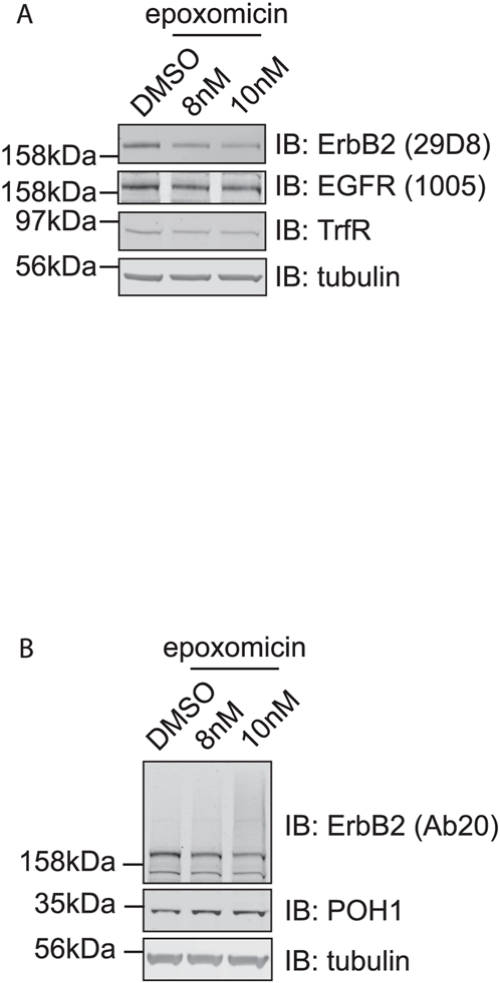
Chronic treatment with a proteasome inhibitor replicates effects of POH1 depletion on ErbB2. HeLa cells were incubated with epoxomicin (8 nM or 10 nM) or DMSO for 48 hours (fresh inhibitors were applied at 24 hours). Cell lysates obtained by “hot lysis” were subjected to SDS-PAGE followed by immunoblotting with ErbB2, EGFR, TrfR, POH1, and tubulin antibodies. Epoxomicin treatment resulted in the apparent loss of ErbB2 and concomitant appearance of a high molecular weight smear.

POH1 is a component of the 19S proteasomal lid complex and its knockdown inhibits proteasome activity [20]. Blotting cell lysates for ubiquitin revealed an accumulation of ubiquitinated proteins comparable to that observed in the presence of proteasome inhibitors, however in distinction to acute proteasome inhibition (for 6 hours) free ubiquitin levels were not reduced, but if anything slightly increased (Figure 8). We next asked the question whether we could reproduce the effects of POH1 depletion on ErbB2 levels by chronic inhibition of proteasome activity. We titrated epoxomicin in long-term culture to determine the maximum tolerated dose and analysed the effect on ErbB2 levels. Sustained application of 10 nM epoxomicin recapitulated the selective loss of ErbB2 seen following POH1 knockdown, with minimal effect on EGFR levels (Figure 9A), whilst the extracellular domain antibody, Ab20, again showed a higher molecular weight smear upon proteasome inhibition (Figure 9B).

## Discussion

The degradation rate of activated RTKs may be controlled by the balance of ubiquitination by E3 ligases (such as c-Cbl) and DUBs such as AMSH [21], [22]. ErbB2 is not reduced following indirect activation through stimulation of EGFR, but can be destabilised by CHIP-dependent ubiquitination following dissociation of Hsp90, the target of Geldanamycin [10], [23]. Previous studies have determined the influence of ErbB2 on acute EGFR down-regulation following over-expression of ErbB2 [5], [6], [7]. Here, we have examined the influence of endogenous ErbB2 on endogenous EGFR down-regulation in HeLa cells, for which both receptors are estimated to be expressed at similar levels. In this instance, we clearly show that depletion of ErbB2 does not influence EGFR down-regulation kinetics.

Initially we reasoned that tonic DUB activity may contribute to ErbB2 stability and that we may identify a relevant DUB with a siRNA screen. This predicts that knockdown of a specific DUB would lead to decreased ErbB2 levels due to ubiquitin-dependent degradation. The initial screen using the siGenome pool of 4 oligos per target identified several candidates, but these did not pass the second round of validation i.e.≥two of four On-Target Plus oligos recapitulating the effect.

One DUB, POH1, passed our validation study (4/4 oligos). POH1 is a component of the 19S proteasomal lid complex and has been suggested to couple recycling of ubiquitin to protein degradation [24]. Although we see a high degree of loss of ErbB2 following POH1 knock-down, especially when using an antibody directed at the cytosolic domain, when we assay surface associated ErbB2 with an extra-cellular directed antibody we do not observe a corresponding loss of receptor. Furthermore, in these POH1-depleted cells, Western blotting for ErbB2 with an extracellular domain directed antibody reveals a higher molecular weight smear characteristic of ubiquitinated receptor that is susceptible to USP2-cleavage. Thus, we propose that POH1 is an ErbB2 DUB, which may oppose constitutive ubiquitination of the receptor.

We can see the accumulation of ubiquitinated ErbB2 under conditions where proteasome activity is blocked, either by POH1 knockdown or by epoxomicin. Remarkably, the ubiquitinated ErbB2 is not rapidly down-regulated by the lysosomal pathway as happens for example with a truncated EGFR fused to a single linear ubiquitin [25], [26]. Our data however fits with the idea of a specific domain within ErbB2 that restricts ligand-dependent degradation independently of ubiquitination status [27].

Our study also indicates that the proteasomal pathway is unlikely to be a major degradative pathway for ErbB2 under steady state conditions as we do not see any accumulation of ErbB2 upon POH1 knockdown as measured by FACS analysis. Rather we suggest that our results indicate a novel role for POH1 in deubiquitinating ErbB2 that may in fact rescue it from proteasomal and lysosomal degradation. It is also possible that POH1 may de-ubiquitinate EGFR under steady-state conditions, in which case the receptor will be rescued from degradation in the lysosomal pathway. This is consistent with our observation of enhanced EGFR degradation rate following POH1 knock-down (Figure 6) and chimes with reports that both EGFR and TrkA receptor can be deubiquitinated by proteasome associated activity [28], [29]. Note that this enhanced turnover of EGFR is unlikely due to alterations in ErbB2 levels, as direct depletion of ErbB2 did not affect the downregulation of EGFR (Figure 2).

Using doses of proteasome inhibitors in which HeLa cells remain viable, we found that we could recapitulate the apparent loss of ErbB2 and the selectivity for ErbB2 compared with other RTKs (EGFR and Met), once again accumulating a higher molecular weight smear as judged by Western blotting. Marx et al. have also recently observed diminution of ErbB2 levels in breast cancer cell lines following application of the proteasome inhibitor Velcade/Bortezomib [30]. However, in their study of cells expressing high levels of ErbB2, they report that proteasome inhibition leads to an intracellular accumulation of remaining ErbB2 consistent with targeting for lysosomal degradation. In contrast, we found that both surface and total levels of ErbB2 are actually little changed in the absence of POH1 activity. We were also unable to rescue or increase ErbB2 levels using a specific inhibitor of lysosomal acidification (concanamycin, data not shown), which is at odds with results obtained with chloroquine in the above study by Marx et al. It is possible that differences in cell lines and disparate expression levels of receptors are responsible for these discrepancies. However this may also suggest that POH1 knockdown does not simply phenocopy proteasome inhibition, a fact that is also indicated by the opposite effects of both treatments on free ubiquitin levels (Figure 8).

A recent study has indicated a synergistic interaction between an ErbB2 directed monoclonal antibody Herceptin/Trastuzumab and Velcade with respect to cell death of tissue cultured breast cancer cells [31]. Further study of this interaction as well as epidemiological data on responsiveness to Velcade and ErbB2 status is clearly warranted.

## Materials and Methods

### Antibodies and other reagents

Rabbit polyclonal anti-Met (C-28) and anti-TrfR (H-300), goat polyclonal (1005) anti-EGFR, FITC conjugated ErbB2 (24D2) and PE conjugated EGFR (528) antibodies were from Santa Cruz. Mouse monoclonal anti-ErbB2 antibody (Ab20) was purchased from Neomarkers. Mouse anti-pMAPK, rabbit anti-ErbB2 (29D8), anti-EGFR (2232), anti-MAPK antibodies were obtained from Cell Signaling. Mouse monoclonal anti-tubulin and rabbit anti-ubiquitin (U5379) were from Sigma. Mouse monoclonal anti-polyubiquitinated proteins (FK1) was from BIOMOL international. Rabbit anti-POH1 was obtained from Zymed laboratories. Rabbit anti-Hrs, anti-AMSH, and anti-STAM were described previously [21], [32], [33]. Secondary donkey anti-mouse and anti-rabbit IRDye (680 and 800 nm) antibodies were obtained from LI-COR. Lactacystin, epoxomicin, and concanamycin were purchased from Calbiochem. DUB siRNA library (siGenome) and individual siRNAs were obtained from Dharmacon.

### Cell culture and RNAi experiments

All tissue culture reagents were from Invitrogen unless specified. HeLa, HEK293T, and A549 cells were cultured at 37°C with 5% CO_2_ in Dulbecco's modified Eagle's medium supplemented with 10% foetal bovine serum and 1% non-essential amino acids. DU145 and SKBr3 cells were grown under the same conditions in RPMI and McCoy's (Sigma) medium respectively. In siRNA mediated knockdown experiments, HeLa cells were treated with siRNA oligos at 40–45 nM using Oligofectamine in the absence of serum. After 4 hours, FBS was added to a final concentration of 10%. siRNA duplexes used are as follows, ErbB2 (sense UGGAAGAGAUCACAGGUUAUU, antisense 5′PUAACCUGUGAUCUCUUCCAUU), POH1 OL1 (sense GAACAAGUCUAUAUCUCUUUU, antisense 5′PAAGAGAUAUAGACUUGUUCUU), POH1 OL2 (sense GGCAUUAAUUCAUGGACUAUU, antisense 5′PUAGUCCAUGAAUUAAUGCCUU), POH1 OL3 (sense AGAGUUGGAUGGAAGGUUUUU, antisense 5′PAAACCUUCCAUCCAACUCUUU), POH1 OL4 (sense GAUGGUUGUUGGUUGGUAUUU, antisense 5′PAUACCAACCAACAACCAUCUU).

### Estimation of ErbB2 receptor levels in HeLa cells in comparison to SKBr3 cells

Two dishes of SKBr3 and HeLa cells were set up in parallel. Two days later, one dish each was trypsinised and cells counted by haemocytometer, while each parallel dish was lysed in NP-40 lysis buffer (0.5% Nonidet P-40, 25 mM Tris/HCl pH7.5, 100 mM NaCl, 50 mM NaF, protease inhibitors) on ice, and the samples precleared by centrifugation. The amount of protein per cell was established by BCA assay and samples were analysed by SDS-PAGE followed by Western blot.

### Cell lysis and immunoblotting

Cells were lysed with either NP-40 lysis buffer on ice or SDS “hot lysis buffer” (1% SDS, 1 mM EDTA, and 50 mM NaF) heated to 110°C. Cell lysates were separated by SDS-PAGE followed by immunoblotting, and finally analysed with a LI-COR Odyssey 2.1 system.

### siRNA DUB screen

HeLa cells were grown in 6-well plates to 30–50% confluency and then transfected with DUB specific siRNA oligos at 40 nM, using Oligofectamine (Invitrogen). After 72 hours, cells were lysed with NP-40 lysis buffer. Lysates were subjected to SDS-PAGE followed by immunoblotting with ErbB2 (29D8) and tubulin antibodies. ErbB2 bands were quantified with the LI-COR Odyssey 2.1 system and ImageJ and normalized to tubulin.

### Real time PCR

HeLa cells were treated with control reagent or POH1 siRNA for 24 or 48 hours. RNA was extracted using a Qiagen RNAeasy kit. cDNA was prepared with a QuantiTect® reverse transcription kit (Qiagen). RT-PCR reactions were set up using DyNAmo HS SYBR Green qPCR kit (Finnzymes) and run on the BioRad iQ5 system according to the manufacturer's instructions. Experimental data for EGFR and ErbB2 were normalized to actin.

### Flow cytometry

HeLa cells were treated with control reagent or POH1 siRNA for 48 hours. Cells were detached by incubation at 37°C in PBS supplemented with EDTA. One million cells were used per reaction, which were first washed with PBS supplemented with 0.1% BSA and 0.05% sodium azide (PBS/B/A) and then incubated with Phycoerythrin conjugated EGFR and fluorescein isothiocyanate (FITC) conjugated ErbB2 antibodies (Santa Cruz) for 30 minutes in the same buffer on ice. Cells were washed once and then resuspended in 0.5 ml of PBS/B/A, finally analysed by flow cytometry using a FACScan cytometer.

### In vitro deubiquitination assay with USP2 catalytic domain

HeLa cells were treated with POH1 siRNA or control reagent for 48 hours and lysed in RIPA buffer (10 mM Tris, pH 7.5, 100 mM NaCl, 1% NP-40, 0.1% SDS, 1% sodium deoxycholate, and 50 mM NaF) supplemented with protease and phosphatase inhibitors, and 10 mM *N*-ethylmaleimide (NEM). Cell lysates were cleared by centrifugation, equal amounts for each condition were incubated with prewashed protein G agarose and anti-ErbB2 antibody (Ab5, extracellular epitope, Calbiochem) for 2 hours at 4°C. Beads were washed 3 times with RIPA buffer, twice with deubiquitination assay buffer (50 mM HEPES pH 7.3, 0.5 mM EDTA) and finally resuspended in 250 µl of deubiquitination assay buffer with 2 mM DTT. Samples with or without USP2 catalytic domain (100 nM; BIOMOL, UW9850) were incubated in a thermoshaker for 8 hours (37°C, 1000 rpm). Beads were then washed twice with 10 mM Tris pH 7.5, proteins eluted with SDS sample buffer and samples analyzed by immunoblotting with anti-ErbB2 antibodies recognizing extracellular (Ab20, Neomarkers) and intracellular (29D8, Cell signaling) epitopes.

## References

[1] Yarden Y, Sliwkowski MX (2001). Untangling the ErbB signalling network.. Nat Rev Mol Cell Biol.

[2] Sorkin A, Goh LK (2008). Endocytosis and intracellular trafficking of ErbBs.. Exp Cell Res.

[3] Urbe S (2005). Ubiquitin and endocytic protein sorting.. Essays Biochem.

[4] Lenferink AE, Pinkas-Kramarski R, van de Poll ML, van Vugt MJ, Klapper LN (1998). Differential endocytic routing of homo- and hetero-dimeric ErbB tyrosine kinases confers signaling superiority to receptor heterodimers.. Embo J.

[5] Wang Z, Zhang L, Yeung TK, Chen X (1999). Endocytosis deficiency of epidermal growth factor (EGF) receptor-ErbB2 heterodimers in response to EGF stimulation.. Mol Biol Cell.

[6] Haslekas C, Breen K, Pedersen KW, Johannessen LE, Stang E (2005). The inhibitory effect of ErbB2 on epidermal growth factor-induced formation of clathrin-coated pits correlates with retention of epidermal growth factor receptor-ErbB2 oligomeric complexes at the plasma membrane.. Mol Biol Cell.

[7] Worthylake R, Opresko LK, Wiley HS (1999). ErbB-2 amplification inhibits down-regulation and induces constitutive activation of both ErbB-2 and epidermal growth factor receptors.. J Biol Chem.

[8] Finley D, Ciechanover A, Varshavsky A (2004). Ubiquitin as a central cellular regulator.. Cell.

[9] Mimnaugh EG, Chavany C, Neckers L (1996). Polyubiquitination and proteasomal degradation of the p185c-erbB-2 receptor protein-tyrosine kinase induced by geldanamycin.. J Biol Chem.

[10] Zhou P, Fernandes N, Dodge IL, Reddi AL, Rao N (2003). ErbB2 degradation mediated by the co-chaperone protein CHIP.. J Biol Chem.

[11] Citri A, Alroy I, Lavi S, Rubin C, Xu W (2002). Drug-induced ubiquitylation and degradation of ErbB receptor tyrosine kinases: implications for cancer therapy.. Embo J.

[12] Austin CD, De Maziere AM, Pisacane PI, van Dijk SM, Eigenbrot C (2004). Endocytosis and sorting of ErbB2 and the site of action of cancer therapeutics trastuzumab and geldanamycin.. Mol Biol Cell.

[13] Lerdrup M, Hommelgaard AM, Grandal M, van Deurs B (2006). Geldanamycin stimulates internalization of ErbB2 in a proteasome-dependent way.. J Cell Sci.

[14] Pedersen NM, Madshus IH, Haslekas C, Stang E (2008). Geldanamycin-induced down-regulation of ErbB2 from the plasma membrane is clathrin dependent but proteasomal activity independent.. Mol Cancer Res.

[15] Nijman SM, Luna-Vargas MP, Velds A, Brummelkamp TR, Dirac AM (2005). A genomic and functional inventory of deubiquitinating enzymes.. Cell.

[16] Daviet L, Colland F (2008). Targeting ubiquitin specific proteases for drug discovery.. Biochimie.

[17] Yang S, Raymond-Stintz MA, Ying W, Zhang J, Lidke DS (2007). Mapping ErbB receptors on breast cancer cell membranes during signal transduction.. J Cell Sci.

[18] Berkers JA, van Bergen en Henegouwen PM, Boonstra J (1991). Three classes of epidermal growth factor receptors on HeLa cells.. J Biol Chem.

[19] Polo S, Sigismund S, Faretta M, Guidi M, Capua MR (2002). A single motif responsible for ubiquitin recognition and monoubiquitination in endocytic proteins.. Nature.

[20] Koulich E, Li X, Demartino GN (2008). Relative Structural and Functional Roles of Multiple Deubiquitylating Proteins Associated with Mammalian 26S Proteasome.. Mol Biol Cell.

[21] McCullough J, Clague MJ, Urbe S (2004). AMSH is an endosome-associated ubiquitin isopeptidase.. J Cell Biol.

[22] Clague MJ, Urbe S (2006). Endocytosis: the DUB version.. Trends Cell Biol.

[23] Xu W, Marcu M, Yuan X, Mimnaugh E, Patterson C (2002). Chaperone-dependent E3 ubiquitin ligase CHIP mediates a degradative pathway for c-ErbB2/Neu.. Proc Natl Acad Sci U S A.

[24] Verma R, Aravind L, Oania R, McDonald WH, Yates JR (2002). Role of Rpn11 metalloprotease in deubiquitination and degradation by the 26S proteasome.. Science.

[25] Mosesson Y, Shtiegman K, Katz M, Zwang Y, Vereb G (2003). Endocytosis of receptor tyrosine kinases is driven by monoubiquitylation, not polyubiquitylation.. J Biol Chem.

[26] Haglund K, Sigismund S, Polo S, Szymkiewicz I, Di Fiore PP (2003). Multiple monoubiquitination of RTKs is sufficient for their endocytosis and degradation.. Nat Cell Biol.

[27] Shen F, Lin Q, Childress C, Yang W (2008). Identification of the domain in ErbB2 that restricts ligand-induced degradation.. Cell Signal.

[28] Geetha T, Wooten MW (2008). TrkA receptor endolysosomal degradation is both ubiquitin and proteasome dependent.. Traffic.

[29] Alwan HA, van Leeuwen JE (2007). UBPY-mediated epidermal growth factor receptor (EGFR) de-ubiquitination promotes EGFR degradation.. J Biol Chem.

[30] Marx C, Yau C, Banwait S, Zhou Y, Scott GK (2007). Proteasome-regulated ERBB2 and estrogen receptor pathways in breast cancer.. Mol Pharmacol.

[31] Cardoso F, Durbecq V, Laes JF, Badran B, Lagneaux L (2006). Bortezomib (PS-341, Velcade) increases the efficacy of trastuzumab (Herceptin) in HER-2-positive breast cancer cells in a synergistic manner.. Mol Cancer Ther.

[32] Urbé S, Mills IG, Stenmark H, Kitamura N, Clague MJ (2000). Endosomal localization and receptor dynamics determine tyrosine phosphorylation of hepatocyte growth factor-regulated tyrosine kinase substrate.. Mol Cell Biol.

[33] Row PE, Clague MJ, Urbe S (2005). Growth factors induce differential phosphorylation profiles of the Hrs-Stam complex: a common node in signaling networks with signal specific properties.. Biochem J.

